# An analysis of the main driving factors of renewable energy consumption in the European Union

**DOI:** 10.1007/s11356-022-18715-z

**Published:** 2022-01-19

**Authors:** José Antonio Camacho Ballesta, Lucas da Silva Almeida, Mercedes Rodríguez

**Affiliations:** 1 Department of International and Spanish Economics, Faculty of Economics and Business, University of Granada, 18071 Granada, Spain; 2 Institute of Regional Development, University of Granada, 18071 Granada, Spain; 3 University Center Maria Milza, Governador Mangabeira, 44350 Brazil

**Keywords:** Renewable energy, Economic factors, Social factors, Energy transition, Data panel, European Union, O52, Q20, Q42

## Abstract

Climate change is a major global concern closely related to the strategies aimed at reducing energy consumption and increasing energy efficiency. Over the last decades, the interest in the development of renewable energy (RE) has grown exponentially. In the case of the European Union (EU), the Renewable Energy Directive sets rules to achieve a 32% of total energy consumption to be covered through RE by 2030. In order to achieve this goal, it is important to know what are the main driving factors of RE consumption (REC). This study aims to analyze the impact of economic and social factors on the share of REC in total energy consumption in the EU over the period 2001–2015. For doing so, we estimate a Panel Corrected Standard Error (PCSE) model. The results obtained show that economic factors have a negative effect on REC. In contrast, social factors like education exert a positive effect. This suggests that it is necessary to adopt a holistic approach that includes not only economic but also social aspects in order to foster REC.

## Introduction

Climate change is a major global concern. The strategy to reverse the current situation is closely related to the way in which energy is generated and consumed. In this regard, renewable energy (RE) is a key element that contributes, not only to the conservation of the environment, but also to economic and social development (Saint Akadiri et al. 2019).

Concerning the production of RE, the data from the International Energy Agency (IEA) reveal that the share of primary energy from renewable sources in the world has hardly changed over the last decades. Thus, in 1971 RE accounted for 13.1% of primary energy. In 2018 the share was almost identical: 13.8% (IEA 2020a). This slow pace of change is explained by a wide range of problems from the inefficient way in which some people and firms use energy to the lack of information and/or knowledge on the importance of clean energy, the existence of market failures or the accessibility to raw materials (Painuly 2001; Owen 2006; Verbruggen et al. 2010; Sen and Ganguly 2017).

Regarding the consumption of RE, according to the global renewable community of actors REN21, in 2019 renewable energy consumption (REC) accounted for 19.9% of world total final energy consumption (TFEC) (REN21 2021). REN21 differentiates between modern renewable energies and traditional biomass. Thus, as of 2019, modern renewable energies accounted for 11.2% of TFEC, 2.5 percentage points more than a decade earlier. Within modern renewable energies, the most important one was renewable electricity, which represented 6% of TFEC, followed by renewable heat (4.2%) and transport biofuels (1%) (REN21 2021).

The European Union (EU) is aware of the importance of REC as a facilitating tool for meeting the sustainable development goals (SDGs) proposed by the United Nations (UN), and, in particular, for the achievement of an affordable and non-polluting energy system. Beyond contributing to the reduction of greenhouse gas emissions and to the diversification of energy supply, REC is also a tool to reduce the dependence on non-EU fossil fuels markets, considered to be volatile and unreliable (European Parliament 2019). For 2030 the initial target of 27% of total energy consumption to be covered through RE has been rised to a percentage of at least 32%, as reported in Directive (EU) 2018/2001 (European Union 2018). Meeting this objective is a major challenge. According to the data published by Eurostat (2021), in 2019 REC represented 19.7% of the energy consumed in the EU27. There are, however, important differences across countries. Thus, for one part we find countries that more than double the EU27 average, like Sweden (56.4%) or Finland (43.1%). For the other part, there are countries with a very low share of REC, like Luxembourg (7%), the Netherlands (8.8%) or Malta (8.5%).

Direct policy support, in the form of economic, administrative, financial and regulatory support measures, is essential for the deployment of RE. In 2015, the last year analyzed in our study, more than 1300 support measures for the development of RE were in place in the EU countries. Feed-in-tariff and feed-in-premiums were the main supporting schemes in the electricity sector, and they were applied in 24 EU countries (Banja et al. 2018). In recent years the interest is shifting away towards auctions, as they lead to lower prices and higher realization rates (REN21 2021).

In their seminal study on the relationship between gross energy consumption and gross domestic product (GDP), Kraft and Kraft (1978) found a bidirectional causality between these two variables. Following this line of analysis, different studies have examined the link between REC and other variables, not only, related to economic growth like GDP but also related to trade, foreign direct investment (FDI) or CO_2_ emissions. The results of these studies are various and varied. Although a growing strand of the literature deals with REC, very few papers have analyzed the driving factors of the share of REC in total energy consumption (Ito 2017; Attiaoui et al. 2017; Mele 2019; Toumi and Toumi 2019; Ergun et al. 2019; Anton and Afloarei Nucu 2020; Khan et al. 2020; Khribich et al. 2021; Marra and Colantonio 2021; Lei et al. 2021). This paper examines the main driving factors of the share of REC in the EU over the period 2001–2015 by estimating a Panel Corrected Standard Errors (PCSE) model. It contributes to the extant literature in two main ways. First, we examine the impact of both economic and social factors. A large body of the literature on the driving forces of REC includes economic variables. However, there is a lack of evidence on the impact of social factors, especially in high-income countries. Among the factors that affect RE uptake we can highlight political conditions and governance, as they directly affect public support of RE. For this reason, within our group of social factors, in addition to indices on life expectancy and education, we include an index of governance. This index is constructed by combining six different indicators. Second, we carry out our analysis at the world level and at the EU level in order to identify the existence of differential features in the EU. Many of the drivers of RE deployment depend on national contexts. In particular demographics and socio-economic characteristics shape the pace and nature of REC. Thus, we can expect the same factor to have a different effect depending on the region or country examined.

The article is organized as follows. The second section presents a brief literature review on those works dealing with REC. The third section describes the data and methodology employed. Next, we discuss the main results. Finally, we summarize the main findings and make some policy recommendations.

## The driving forces of renewable energy consumption: a review of the literature

To know in depth the determinants of REC is key when designing efficient energy strategies. The literature dealing with the driving forces of REC employ four main variables to proxy REC, namely, the total renewable energy consumption (TREC) (Apergis and Payne 2010a; Apergis et al. 2010; Salim and Shafiei 2014; Salim et al. 2014; Lin and Moubarak 2014; Ben Aïssa et al. 2014; Ben Jebli 2016; Doytch and Narayan 2016; Saidi and Ben Mbarek 2016; Cherni and Jouini 2017; Chen 2018; Rasoulinezhad and Saboori 2018; Marinaş et al. 2018; Nguyen and Kakinaka 2019; Eren et al. 2019; Olanrewaju et al. 2019; Zhao et al. 2020; Sohail et al. 2021; Baye et al. 2021), the renewable energy consumption per capita (RECpc) (Sadorsky 2009a, b; Salim and Rafiq 2012; Apergis and Payne 2014; Omri et al. 2015; Ben Jebli et al. 2015; Ben Jebli and Ben Youssef 2017; Hashemizadeh et al. 2021), the renewable electric energy consumption (REEC) (Apergis and Payne 2010b, 2011, 2012; Al-Mulali et al. 2013; Sebri and Ben-Salha 2014; Amri 2017, 2019) and the share of renewable energy consumption in total energy consumption (REC%) (Ito 2017; Attiaoui et al. 2017; Mele 2019; Toumi and Toumi 2019; Ergun et al. 2019; Anton and Afloarei Nucu 2020; Khan et al. 2020; Khribich et al. 2021; Marra and Colantonio 2021; Lei et al. 2021). Table 1 presents a summary of the main studies conducted to date.

**Table 1:** Previous studies on the driving forces of REC

Authors	Period	Countries	Methodology	Proxy
				for REC
Al-Mulali et al. (2013)	1980–2009	109 countries	FMOLS	REEC
Amri (2017)	1990–2012	72 countries	GMM; Dynamic panel estimator Blundell-Bond	REEC
Amri (2019)	1990–2012	72 countries	GMM	REEC
Anton and Afloarei Nucu (2020)	1990–2015	28 EU countries	Fixed effects panel model	REC%
Apergis and Payne (2010a)	1992–2007	13 Eurasia countries	VECM; Granger-causality tests	REEC
Apergis and Payne (2010b)	1985–2005	20 OECD countries	VECM; Granger-causality tests	TREC
Apergis and Payne (2011)	1990–2007	80 countries	Panel error correction models	REEC
Apergis and Payne (2012)	1990–2007	80 countries	VECM; causal dynamics	REEC
Apergis and Payne (2014)	1980–2010	7 Central American countries	Long-run cointegration vector using FMOLS	RECpc
Apergis et al. (2010)	1984–2007	19 developed and developing countries	Dynamic error correction model, Granger causality	TREC
Apergis et al. (2018)	1995–2011	42 Sub-Saharan Africa countries	Granger causality tests	REC%
Attiaoui et al. (2017)	1990–2011	22 African countries	ARDL-PMG; Granger causality	REC%
Baye et al. (2021)	1990–2015	32 Sub-Saharan Africa countries	ARDL	TREC
Ben Aïssa et al. (2014)	1980–2008	11 African countries	VECM	TREC
Ben Jebli (2016)	1990–2011	Tunisia	ARDL	TREC
Ben Jebli and Ben Youssef (2017)	1980–2011	Tunisia	VECM; Granger causality tests	RECpc
Ben Jebli et al. (2015)	1980–2010	24 sub-Saharan Africa countries	Granger causality tests	RECpc
Chen (2018)	1996–2013	China	GMM	TREC
Cherni and Jouini (2017)	1990–2015	Tunisia	ARDL and Granger causality tests	TREC
Doytch and Narayan (2016)	1985–2012	74 countries	GMM; Dynamic panel estimator Blundell-Bond	TREC
Eren et al. (2019)	1971–2015	India	Quasi-GLS; Cointegration test; DOLS and Granger causality	TREC
Ergun et al. (2019)	1990–2013	21 African countries	FE and RE GLS	REC%
Hashemizadeh et al. (2021)	1990–2016	20 emerging countries	DKSE; FGLS; PCSE	RECpc
Ito (2017)	2002–2011	42 developing countries	GMM; PMG	REC%
Khan et al. (2020)	1980–2018	192 countries	Panel quantile regression model	REC%
Khribich et al. (2021)	1995–2015	27 countries	Granger causality tests	REC%
Lei et al. (2021)	1990–2019	China	ARDL	REC%
Lin and Moubarak (2014)	1977–2011	China	ARDL	TREC
Marinaş et al. (2018)	1990–2014	10 EU members from CEE	PMG estimator for the error correction models	TREC
Marra and Colantonio (2021)	1990–2015	12 EU countries	PVAR model	REC%
Mele (2019)	1980–2017	Mexico	Multivariate Granger causality tests	REC%
Nguyen and Kakinaka (2019)	1990–2013	107 countries	FMOLS and DOLS	TREC
Olanrewaju et al. (2019)	1990–2015	5 African countries	Pooled OLS, Panel FE and RE	TREC
Omri et al. (2015)	1990–2011	64 countries	Pooled OLS; Panel FE and RE; GMM	RECpc
Rasoulinezhad and Saboori (2018)	1992–2015	CIS region countries	FMOLS; DOLS	TREC
Sadorsky (2009a)	1980–2005	G7 countries	Cointegration of panels; FMOLS; DOLS	RECpc
Sadorsky (2009b)	1994–2003	18 emerging countries	FMOLS; DOLS; OLS	RECpc
Saidi and Ben Mbarek (2016)	1990–2013	9 developed countries	Granger causality; FMOLS; DOLS	TREC
Salim and Rafiq (2012)	1980–2006	6 emerging economies	ARDL; FMOLS; DOLS; and Granger causality tests	RECpc
Salim and Shafiei (2014)	1980–2011	29 OECD countries	Common Correlated Effects	TREC
Salim et al. (2014)	1980–2012	29 OECD countries	PMG; ARDL	TREC
Sebri and Ben-Salha (2014)	1971–2010	3 BRICS countries	ARDL; FMOLS, DOLS	REEC
Sohail et al. (2021)	1985–2019	USA	ARDL; NARDL	TREC
Toumi and Toumi (2019)	1990–2014	Saudi Arabia	NARDL	REC%
Zhao et al. (2020)	1980–2016	China	OLS; FMOLS	TREC

*ARDL* autoregressive distributed lag model, *DOLS* dynamic ordinary lest squares, *DKSE* Driscoll-Kraay standard errors, *FE* fixed effects, *FGLS* feasible generalized least squares, *FMOLS* fully modified least square, *GLS* generalized least squares, *GMM* generalized method of moments, *NARDL* non-linear autoregressive distributed lag model, *OLS* ordinary least squares, *PCSE* Panel Corrected Standard Errors, *PMG* pooled mean group, *PVAR* panel vector autoregressive, *RE* random effects, *VECM* vector correction model

Most of studies employ TREC and include GDP per capita as an explanatory factor that represents income and the level of development. Other common economic explanatory factors are FDI and trade openness (TO). Concerning FDI, Doytch and Narayan (2016) examine the impact of the sectoral distribution of FDI on industrial REC in 74 countries over the period 1985–2012. They find that total FDI encourages industrial REC, but that there are differential impacts at the sectoral level. Thus, while financial service FDI fosters REC manufacturing FDI reduces REC. In their analysis of 21 African countries between 1990 and 2013, Ergun et al. (2019) also find a positive impact of FDI on REC%. However, the study for 192 countries over the period 1980–2018 conducted by Khan et al. (2020) obtains heterogenous results for FDI. Thus, in their panel quantile regression non-significant results at low quantile become positive and significant at higher quantiles. They conclude that FDI reduces REC% in lower countries and increases in higher ones. In contrast, Anton and Afloarei Nucu (2020) find a negative relationship between FDI and REC% in the EU countries during the period 1990–2015, and Lei et al. (2021), in their analysis of China, do not find any link between FDI and REC%. The findings on the relationship between TO and REC are either uniform. For one part, some studies obtain a positive relationship between trade and REC (Sebri and Ben-Salha 2014; Amri 2017, 2019; Rasoulinezhad and Saboori 2018), and, for the other part, other studies show the existence of a negative relationship between TO and REC. Thus, Amri (2017), in his analysis of 72 countries over the period 1990–2012, obtains a positive effect of trade on REEC in all groups of countries. Nonetheless, the shape of the impact differs between developed/industrialized countries and developing/non-industrialized ones: while in developed/industrialized countries the relationship shows a U-shape, in the case of developing/non industrialized countries the relationship has an inverted U shape (Amri 2019; Naqvi et al. 2020). The analysis of 12 countries of the Commonwealth of Independent States (CIS) region between 1992 and 2015 carried out by Rasoulinezhad and Saboori (2018) finds the existence of a bidirectional relationship between TO and renewable energy use. In the case of the BRICS countries, the study for the period 1971–2010 conducted by Sebri and Ben-Salha (2014) shows the existence of a significant effect of TO on REEC. In their analysis of 20 emerging countries between 1990 and 2016, Hashemizadeh et al. (2021) find that TO decreases RECpc. In the same vein, the studies by Ergun et al. (2019) and Khan et al. (2020) obtain a negative impact of TO on REC%. Finally, in their analysis of China, Zhao et al. (2020) find that TO raises non-renewable energy consumption.

Turning to social factors, we have to highlight that the incorporation of this type of factors is less common. We can cite the studies of Apergis et al.(2018), Baye et al. (2021), Ben Jebli (2016), Ergun et al. (2019), Khribich et al. (2021) and Marra and Colantonio (2021). In their examination of a panel of 42 sub-Saharan Africa countries over the period 1995–2011, Apergis et al. (2018) introduce health expenditures as a health indicator. They find a long-run unidirectional causality from REC% to health care expenditures. Focusing of 32 Sub-Saharan Africa countries, Baye et al. (2021) assess, among other variables, the impact of the quality of governance. They find a positive association between TREC and the improvement in the quality of governance. In his study of Tunisia, Ben Jebli (2016) obtains a bi-directional relationship between health and the consumption of combustible renewables. Ergun et al. (2019) examine the impact of the Human Development Index (HDI) and of the level of democracy, proxied by the aggregated weights of political rights and civil liberties ratings, on REC% in Africa. They obtain a negative impact of the HDI on REC%, while the effect of the level of democracy is non-significant. Khribich et al. (2021) construct a social development index using 17 variables on demography, education, health, consumption and IT and research for 27 high-income countries over the period 1995–2015, finding that social development contributes to REC% in the long run but not in the short run. Finally, Marra and Colantonio (2021) examine the impact on REC% in 12 EU countries of several socio-technical aspects, including educational attainment. They conclude that the combination of public awareness and environmental education can help RE deployment.

As was pointed out before, most of analyses employ the total value of renewable energy consumption as dependent variable. We find analyses for individual countries like China (Lin and Moubarak 2014; Chen 2018; Zhao et al. 2020), India (Eren et al. 2019), Tunisia (Ben Jebli 2016; Cherni and Jouini 2017) or the USA (Sohail et al. 2021), as well as studies for groups of countries, like the OECD countries (Apergis and Payne 2010a; Salim and Shafiei 2014; Salim et al. 2014), EU members from CEE (Marinaş et al. 2018), CIS region countries (Rasoulinezhad and Saboori 2018), African countries (Ben Aïssa et al. 2014; Olanrewaju et al. 2019; Baye et al. 2021), developed countries (Saidi and Ben Mbarek 2016) and both developed and developing countries (Apergis et al. 2010; Doytch and Narayan 2016; Nguyen and Kakinaka 2019). Other studies employ RECpc as a proxy of REC. We can cite the analysis of Tunisia conducted by Ben Jebli and Ben Youssef (2017) and studies for different groups of countries like the G7 countries (Sadorsky 2009a), some emerging countries (Sadorsky 2009b; Salim and Rafiq 2012; Hashemizadeh et al. 2021), Central American countries (Apergis and Payne 2014), African countries (Ben Jebli et al. 2015) or 64 high, low and middle-income countries (Omri et al. 2015). As was pointed out before, the number of studies that examine REC in relative terms, that is, as a share of total energy consumption (REC%) is scarcer. Thus, at the individual country level we find recent analyses conducted for Mexico (Mele 2019), Saudi Arabia (Toumi and Toumi 2019) or China (Lei et al. 2021). Other studies focus on African countries (Apergis et al. 2018; Ergun et al. 2019), on developing countries (Ito 2017) on high-income countries (Khribich et al. 2021) and on European countries (Anton and Afloarei Nucu 2020; Marra and Colantonio 2021). As far as our knowledge, the widest study in terms of number of countries covered (192) was carried out by Khan et al. (2020). In this paper we widen the studies for the European countries conducted by Anton and Afloarei Nucu (2020) and Marra and Colantonio (2021) by comparing the results of the EU with those obtained at the world level and by including social factors. In the next section we describe the data and the methodology employed.

## Data and methodology

### Data

A total of 176 countries over the period 2001–2015 were examined. As mentioned before, we compare the results for the EU countries with those obtained for the whole panel of countries. Within the EU, we include the 28 countries that formed the EU in 2015, namely, Austria, Belgium, Bulgaria, Croatia, Republic of Cyprus, Czech Republic, Denmark, Estonia, Finland, France, Germany, Greece, Hungary, Ireland, Italy, Latvia, Lithuania, Luxembourg, Malta, Netherlands, Poland, Portugal, Romania, Slovakia, Slovenia, Spain Sweden and the UK. The list of countries examined is presented in Appendix Table 9.

The dependent variable (REC%) is defined as the share of REC in total energy consumption. It was obtained from the World Development Indicators Database (World Bank 2019a).

We included three economic explanatory factors: gross domestic product per capita (GDPpc), foreign direct invesstment (FDI) and trade openness (TO) and three social explanatory factors: an education index (EI), a life expectancy index (LEI) and a governance index (GI). GDPpc captures economic growth and income distribution, and it is obtained by dividing the gross domestic product by population. In order to proxy economic growth in real terms, it is valued at constant prices of 2010 (World Bank 2019a). The second economic explanatory variable, FDI, is defined as the net investment inputs to acquire a lasting management interest (10% or more of the voting stock) in a company operating in an economy other than that of the investor. It is the sum of equity capital, reinvestment of earnings, other long-term capital and short-term capital as shown in the balance of payments (World Bank 2019a). Finally, the third economic explanatory factor is TO. Although there are different forms of measuring TO (Yanikkaya 2003), in this study we adopt the definition of the World Bank, and we define it as the share of trade (sum of imports and exports of goods and services) in GDP (World Bank 2019a).

In addition to the economic variables, this study takes into consideration three social variables. The HDI developed by the United Nations Development Program (UNDP) measures three aspects: access to knowledge, a long and healthy life and a decent standard of living. The latter aspect is proxied by GDPpc. Our first two social explanatory factors are the two first components of the HDI, namely, the EI and LEI. The third social explanatory factor is a governance index (GI). The GI is a multidimensional index proposed by Kaufmann et al. (2010) and published by the World Bank (World Bank 2019b). The GI has been used in previous studies on other issues like the relationship between governance quality and net migration flows (Ariu et al. 2016), the link between governance and economic growth (Emara and Chiu 2016), the impact of terrorism on governance (Asongu and Nwachukwu 2017) or the effect of the use of mobile telephony in promoting good governance (Asongu et al. 2018). In our case the GI is constructed by combining the six following indicators:

- Voice and accountability: they measure the extent to which the citizens of a country can participate in the selection of their government, as well as freedom of expression, freedom of association and a free means.
- Political stability and absence of violence: it measures the likelihood of the government to be destabilized by unconstitutional or violent means, including terrorism.
- Government effectiveness: it measures the quality of public services, the capacity of the public administration and its independence from political pressures as well as the quality of policy-making.
- Regulatory quality: it measures the government’s ability to provide sound policies and regulations that enable and promote private sector development.
- Rule of law: it measures the extent to which agents trust and respect the rules of society, including the quality of performance of contracts and property rights, the police and courts, as well as the likelihood of crime and violence.
- Control of corruption: it measures the extent to which public power is exercised to make private gains, including all forms of corruption, both small and large, as well as the “capture” of the state by elites and private interests.

To combine the six indicators, we conducted a Principal Component Analysis (PCA). This technique allows to reduce a set of correlated variables in a smaller group of uncorrelated variables, which are called components (Hair et al. 1999). The components are independent of each other and are a linear combination of the original variables. One of the main advantages of PCA is that it solves the problem of implicit weighting. The components represent in a decreasing and successive way the percentage of the variance explained from the original set of variables, where the first component represents the highest percentage of variation in the original set of variables, the second captures the largest percentage of the variation that is not explained by the previous one and so on. The results obtained in the Kaiser–Meyer–Olkin test was 0.886, thus allowing to continue with the factorial analysis (Cureton and D’Agostino 2013). The value of the Cronbach’s Alpha for our GI was 0.917, which confirms the adequacy of the index (Vaske et al. 2017).

### Methodology

Following the literature review conducted in the second section, we specify a model based on economic variables and on social variables. From an economic point of view, there is a wide consensus in literature on the fact that economic growth, foreign investment and trade openness influence REC%. However, social factors can make an important difference, especially in those countries with a high level of development. Thus, factors like the levels of education or governance are found to significantly influence REC% (Baye et al. 2021; Marra and Colantonio 2021).

The model estimated can be summarized as follows:

1 $${\mathrm{REC}\%}_{\mathrm{it}}=\beta_0+\beta_1{\mathrm{InGDPpc}}_{\mathrm{it}}+\beta_2{\mathrm{InFDI}}_{\mathrm{it}}+\beta_3{\mathrm{TO}}_{\mathrm{it}}+\beta_4{\mathrm{EI}}_{\mathrm{it}}+\beta_5{\mathrm{LEI}}_{\mathrm{it}}+\beta_6{\mathrm{GI}}_{\mathrm{it}}+{\mathrm e}_{\mathrm{it}}$$

where, *i* denotes the country and *t* the year; β_0_ is the intercept,β_1_, β_2_, β_3_, β_4_, β_5_, and β_6_ are the long-run elasticities of dependent variable; *e* denotes the error terms. REC% is the variable related to share of REC, GDPpc is the gross domestic product per capita, FDI is the foreign direct investment, TO is the trade openness, EI is the education index, LEI is the life expectancy index and GI is the governance index.

Three types of econometric techniques are employed: Ordinary Least Squares (OLS), Feasible Generalized Least Square (FGLS) and Panel Corrected Standard Error (PCSE). According to Gujarati and Porter (2010), when information from the same cross-sectional units exists over time it is possible to design models where the combination of both types of data is used, which can be estimated through panel data techniques. Wooldridge (2010) points out that one of the main advantages of using panel data is that they allow to classify the non-observable factors that influence the dependent variable into two types: those that are constant and those that vary over time (Arellano and Bover 1990; Wooldridge 2002; Plümper et al. 2005). Thus, panel data models can be estimated by using fixed effects or random effects. The fixed effects model considers that differences between units can be captured by differences in the intercept, which in turn implies that each intercept should be estimated. In contrast, the random effects model assumes that each cross-sectional unit has a different intercept. To choose between fixed effects and random effects the Hausman test is commonly employed (Hausman 1978). In spite of its advantages, panel data often present problems of serial autocorrelation, heteroscedasticity and even contemporary correlation (Canarella and Gasparyan 2008). According to Jönsson (2005) these problems arise when disturbances are dependent on the cross section, and these problems can be solved by applying a FGLS model (Parks 1967). However, as Beck and Katz (1995), highlight, the FGLS model produces coefficients whose standard errors are underestimated. In contrast, the PCSE model corrects the presence of serial autocorrelation, heteroscedasticity and even contemporary correlation, with accurate standard error estimates and little or no loss of efficiency compared to FGLS. To assess the independence between errors and the existence of a distribution with constant variance, the Wooldridge test (Wooldridge 2002) and the modified Wald test (Greene 2012) are conducted.

## Results and discussions

Before entering into the results of the model, in Table 2 we compare the share of REC in total energy consumption in the different regions of the world in the beginning and at the end of the period analyzed and in 2018. As the most recent data provided by the World Bank (World Bank 2021) refer to 2015, in order to capture recent changes in REC% we estimated this variable using the data published by the IEA (IEA 2020b).

**Table 2:** Share of REC in total energy consumption by region, 2001–2015–2018

Region	2001	2015	2018*
East Asia and Pacific	21.6	13.9	22.0
Europe and Central Asia	7.6	13.1	23.8
European Union	8.9	17.6	19.6
Latin America and Caribbean	27.8	27.6	34.4
Middle East and North Africa	2.2	1.6	3.1
North America	6.4	10.2	23.1
South Asia	53.0	38.3	38.7
Sub-Saharan Africa	72.9	70.1	55.7
World	17.6	18.1	16.9

Note: *Own estimation from IEA

Source: World Bank (2021) and (IEA, 2020b)

As can be noted, REC% substantially increased over the period 2001–2018 in the EU. Nonetheless, despite the fact that the EU has a technological development level high enough for a strong production and investment on REC (Nicolini and Tavoni 2017; Lilliestam et al. 2019), its share was below the world average in all years. In contrast, regions such as sub-Saharan Africa, South Asia or Latin America and the Caribbean, which report the highest values of REC%, are made up of developing or underdeveloped countries whose production of RE is based on traditional technologies, such as large hydroelectric dams and traditional biomass combustion (Ergun et al. 2019; Baye et al. 2021). Developing and underdeveloped countries outweighed developed countries in REC. Thus, according to the last report of REN21 (REN21 2021), if we compare the evolution of REC over the period 2007–2017 in the group of 37 high-developed member countries of the Organization for Economic Cooperation and Development (OECD) and in the group of non-OECD countries, the growth was substantially higher in non-OECD than in OECD countries (68% compared to 42%). However, if we express REC as a share of TFEC, the growth was lower in non-OECD countries than in OECD countries (29% compared to 44%). It is also necessary to highlight that RE investment varies across regions and countries. Thus, in 2019 considering all financing of RE capacity (excluding hydropower larger than 50 MW) China was the largest investor (30%), followed by the USA (20%), Europe (19%) and Asia-Oceania (16% excluding China and India). In contrast, the smallest shares were reported by Africa and the Middle East (5%), the Americas (4% excluding Brazil and the USA), India (3%) and Brazil (4%) (REN21 2021). These differences can be explained by the stronger economic growth and the improvements in energy access experienced by non-OECD countries and confirm the results of those studies on the renewable energy environment Kuznets curve (RKC). For instance, in their analysis of RKC by income groups, Naqvi et al. (2020) find a U-shaped RKC for high-income countries while in low-income countries the RKC shows an inverted U-shape.

In Table 3, we present the descriptive statistics for the independent and the dependent variables of our model computed for the period 2001–2015 both at the world and at the EU level.

**Table 3:** Descriptive statistics

	Variable	Mean	Std. dev	Min	Max
World	REC%	29.930	30.021	0.000	98.343
	lnGDPpc	8.574	1.529	5.267	12.136
	lnFDI	20.303	2.640	10.361	27.322
	TO	93.541	59.296	0.167	860.800
	EI	60.424	18.159	12.000	94.100
	LEI	75.772	14.101	30.200	98.200
	GI	27.475	15.169	0.000	56.084
European Union	REC%	15.109	11.454	0.000	53.248
	lnGDPpc	10.141	0.687	8.340	11.626
	lnFDI	22.669	1.832	14.509	27.322
	TO	114.405	62.863	45.609	416.389
	EI	81.185	6.459	65.700	94.100
	LEI	89.120	4.767	77.100	96.900
	GI	45.378	6.760	28.335	56.084

In the case of our dependent variable, REC%, as was pointed out before, the average share of RE in total energy consumption at the world level almost double the average share of RE in total energy consumption at the EU level over the period analyzed. The stronger level of economic and social development of the EU is confirmed by the higher mean values of all dependent variables. Thus, both GDPpc, FDI and TO are substantially higher among the EU countries than the world average. Concerning social variables, the major difference is found in terms of education and the minor one in terms of life expectancy. Obviously, the EU is a more homogeneous area than the world so differences across countries are lower within the EU, as confirmed by the lower values of standard deviations. The sole exception is found in the case of TO, where the standard deviation is higher at the EU level than at the world level.

Once examined the descriptive statistics, Table 4 presents the correlation matrix among the variables under study, distinguishing again between the world and the EU. The preliminary analysis of correlation coefficients reveals the existence of some differential features in the EU compared to the world average. Overall, the relationship between the economic variables and REC% is negative and significant. We have to note that the correlation coefficient between REC% and lnGDP is particularly lower in the EU. Regarding social variables, there are important differences between the world and the EU. Thus, whereas the sign of education is positive in the EU, the correlation coefficient between REC% and the EI is negative at the world level. In the case of the governance index, in the EU the relationship between the GI and REC% is weaker compared to the correlation coefficient at the world level.

**Table 4:** Correlation matrix

	Variable	REC%	lnGDPpc	lnFDI	TO	EI	LEI	GI
World	REC%	1						
	lnGDPpc	− 0.693***	1					
	lnFDI	− 0.312***	0.529***	1				
	TO	− 0.309***	0.303***	0.091***	1			
	EI	− 0.637***	0.824***	0.531***	0.252***	1		
	LEI	− 0.677***	0.794***	0.513***	0.242***	0.797***	1	
	GI	− 0.473***	0.809***	0.402***	0.302***	0.725***	0.695***	1
European Union	REC%	1						
	lnGDPpc	− 0.102**	1					
	lnFDI	− 0.347***	0.495***	1				
	TO	− 0.296***	0.187***	− 0.031	1			
	EI	0.180***	0.380***	0.259***	0.003	1		
	LEI	− 0.086*	0.813***	0.471***	0.005	0.234***	1	
	GI	− 0.011	0.832***	0.394***	0.255***	0.477***	0.558***	1

***p ≤ 0.001; **p ≤ 0.005; * *p* ≤ 0.01

As it is widely known, the analysis of correlation coefficients disregards a number of factors such as heteroscedasticity or autocorrelation. To go deeper into the effect of economic and social factors and into the differential features of the EU, we estimate the model depicted in Eq. (1) by using three different econometric techniques: OLS, FGLS and PCSE. The model is estimated both for the world and for the European Union.

As was noted in the methodology sub-section, to adequately estimate the models it is necessary to conduct some tests. The Hausman test (Hausman 1978) is employed to choose between the fixed effects and the random effects model. The null hypothesis is rejected so the fixed-effects models is preferable. Once selected the fixed effects model, two specification tests were conducted, the Wooldridge test (Wooldridge 2002) and the modified Wald test (Greene 2012). The Woolridge test rejects the null hypothesis of no serial correlation and the modified Wald test rejects the null hypothesis of homoscedasticity. Table 5 summarizes the results of the Hausman test and of the two specification tests.

**Table 5:** Results of the specification tests

	World		European Union	
Hausman test	Chi2	Prob	Chi2	Prob
	− 43.44	0.000	− 0.20	0.000
Wooldridge test	*F*	Prob	*F*	Prob
	85.996	0.000	71.27	0.000
Modified Wald test	Chi2	Prob	Chi2	Prob
	640,000.00	0.000	2550.00	0.000

Given the presence of autocorrelation and heteroscedasticity, the FGLS is a suitable model, as it is robust in this case. The estimation results are summarized in Table 6. Additionally, the robustness of the results is tested by estimating the models including only the group of economic variables (Table 7) and only the group of social variables (Table 8).

**Table 6:** Regression results with economic and social variables

Variable	World			European Union		
	OLS	FGLS	PCSE	OLS	FGLS	PCSE
lnGDPpc	− 10.549*	− 9.714*	− 8.275*	− 3.254	− 5.299*	− 5.942*
	(0.626)	(0.453)	(0.717)	(2.051)	(1.405)	(1.806)
lnFDI	1.035*	0.073***	0.176**	− 3.052*	− 0.276*	− 0.515*
	(0.199)	(0.042)	(0.082)	(0.324)	(0.100)	(0.155)
TO	− 0.065*	− 0.004	− 0.021*	− 0.063*	− 0.005	− 0.039*
	(0.007)	(0.003)	(0.005)	(0.008)	(0.006)	(0.007)
EI	− 0.330*	0.012	− 0.134*	0.285*	0.566*	0.507*
	(0.044)	(0.030)	(0.048)	(0.090)	(0.063)	(0.089)
LEI	− 0.797*	− 0.510*	− 0.606*	0.134	0.363*	0.393***
	(0.052)	(0.048)	(0.064)	(0.199)	(0.144)	(0.212)
GI	0.658*	0.039	0.190*	0.539*	− 0.133	0.345**
	(0.046)	(0.027)	(0.044)	(0.149)	(0.095)	(0.139)
Intercept	170.356*	149.136*	152.024*	65.135*	1.045	− 0.478
	(4.021)	(2.769)	(4.040)	(12.076)	(7.882)	(12.256)
*N*	2277	2277	2277	364	364	364
Groups		176	176		28	28
Wald Chi^2^		2857.99	1447.97		141.57	93.09
Chi^2^ prob		0.000	0.000		0.000	0.000
*R*^2^	0.594		0.599	0.318		0.360

***p ≤ 0.10; ** *p* ≤ 0.05; * *p* ≤ 0.01; standard errors in parentheses

**Table 7:** Regression results with economic variables

Variable	World			European Union		
	OLS	FGLS	PCSE	OLS	FGLS	PCSE
lnGDPpc	− 13.810*	− 11.840*	− 12.480*	2.562*	− 2.338*	1.258
	(0.354)	(0.266)	(0.397)	(0.870)	(0.754)	(1.002)
lnFDI	0.373***	0.052	0.126***	− 2.678*	− 0.145	− 0.234*
	(0.196)	(0.032)	(0.068)	(0.321)	(0.097)	(− 0.135)
TO	− 0.060*	− 0.002	− 0.017*	− 0.058*	− 0.008	− 0.047*
	(0.008)	(0.002)	(0.005)	(0.008)	(0.006)	(− 0.008)
Intercept	147.270*	128.901*	138.782*	56.325*	40.108*	14.311
	(3.466)	(2.497)	(3.519)	(8.042)	(7.248)	(9.482)
*N*	2554	2554	2554	390	390	390
Groups		184	184		28	28
Wald Chi^2^		2057.62	1111.80		20.30	37.98
Chi^2^ prob		0.000	0.000		0.000	0.000
*R*^2^	0.496		0.539	0.223		0.292

***p ≤ 0.10; ** *p* ≤ 0.05; * *p* ≤ 0.01; standard errors in parentheses

**Table 8:** Regression results with social variables

Variable	World			European Union		
	OLS	FGLS	PCSE	OLS	FGLS	PCSE
EI	− 0.538*	− 0.186*	− 0.281*	0.377*	0.114**	0.270*
	(0.042)	(0.029)	(0.043)	(0.103)	(0.046)	(0.074)
LEI	− 1.071*	− 0.930*	− 0.920*	− 0.306**	1.011*	0.569*
	(0.052)	(0.045)	(0.060)	(0.145)	(0.137)	(0.174)
GI	0.240*	− 0.113*	− 0.029	− 0.029	− 0.100***	− 0.229*
	(0.042)	(0.025)	(0.038)	(0.113)	(0.053)	(0.080)
Intercept	140.806*	115.158*	120.987*	13.258	− 84.782*	− 46.468*
	(2.663)	(2.561)	(3.313)	(13.218)	(11.334)	(14.318)
*N*	2542	2542	2542	392	392	392
Groups		186	186		28	28
Wald Chi^2^		1736.25	1002.92		103.30	48.92
Chi^2^ prob		0.000	0.000		0.000	0.000
*R*^2^	0.487		0.509	0.047		0.360

***p ≤ 0.10, ** *p* ≤ 0.05, *p ≤ 0.01; standard errors in parentheses

Focusing on the coefficients of the PCSE model, we find that lnGDPpc and TO have negative and statistically significant effects on REC% both at the world and at the EU level. In contrast, GI has a positive and statistically significant effect on REC% both at the world and the EU level. The relationship between education and REC% has different signs at the world and at the EU level. Thus, while the impact of the EI is positive and significant in the EU, it is negative and significant at the world level. The same happens in the case of life expectancy. In contrast, the signs are just the opposite in the case of FDI: the effect is positive and significant at the world level but negative and significant at the EU level. These differences in the sign of some of the variables between the EU and the world can be explained, at least partially, by the high level of development of the EU countries. If we compare the results with those obtained by including only economic variables (Table 7), the effects are the same with the sole exception of the coefficient of LnGDPpc in the EU, that is non-significant. Turning to the coefficients of the model with only social variables (Table 8), the main difference is found in the impact of the GI, which becomes non-significant at the world level and changes its sign at the EU level. We have to note, however, that in the EU, the coefficient for GI in the model with only social variables is significant at the 10% level while in the complete model is significant at the 1% level.

If we compare the results obtained for the EU with those of previous studies on REC in the European countries, we find similarities when the same dependent variable is examined. Thus, the results of the studies by Anton and Afloarei Nucu (2020) and by Marra and Colantonio (2021) that use REC% as dependent variable also show the existence of a negative impact of the level of economic development on REC% in the EU countries. Among other reasons, they argue that a high economic growth results into a higher energy demand that cannot be immediately satisfied by renewable sources. In this sense, the study of Marinaş et al. (2018) that analyzed the impact of GDP on TREC in 10 EU countries from Central and Eastern European countries found that GDP growth has a positive long-term effect on TREC. Regarding FDI, as in our case, Anton and Afloarei Nucu (2020) obtain a negative impact of FDI on REC%. They explain this negative relationship by the fact that FDI fosters investment and innovations by firms, and these cause a reduction in energy use. As was noted before, the coefficient obtained for FDI at the world level is positive. Previous literature on the impact of FDI on REC also obtained mixed results. For instance, the analysis of Doytch and Narayan (2016) find different effects depending on the sector, and the study of Khan et al. (2020) shows the existence of different impacts among countries. As Pacesila et al. (2016) and Gökgöz and Güvercin (2018) highlight, this can be explained by differences in a wide range of factors from the size of the country to its energy policy or energy security.

Entering into the impact of social variables, as was pointed in the review of the literature, the studies incorporating this type of variables are quite scarce. If we focus on the study by Ergun et al. (2019) for African countries that incorporates the HDI and a democracy indicator as explanatory variables of REC%, we can also find some similarities with respect to our results. As Ergun et al. (2019) point out, a higher HDI implies not only a higher income level but also a higher life expectancy and education level. However, they find that a higher HDI reduces REC% in Africa. In our case both life expectancy and education exert a positive impact on REC% in the EU, but the impact is negative at the world level. This differential behavior can be explained by the differences in the development level of EU countries and African countries and more concretely by the “traditional” character of RE production and consumption in the latter ones. For instance, electrification rates are low in African countries, and most of RE used for cooking comes from burning wood. Regarding the level of democracy, Ergun et al. (2019) do not find any statistically significant impact. In their examination of 27 high-income countries, including most of EU countries, Khribich et al. (2021) conclude that social development positively affects REC in the long run. Their definition of social development covers five main aspects: demographic, education, health, consumption and IT and research. Their simulations show that it is necessary an increase in the growth of social development in order to achieve a positive impact of social development in the short run. Finally, in line with our findings, the analysis of 32 Sub-Saharan Africa countries carried out by Baye et al. (2021) finds that the quality of governance has a positive impact on REC%. The effect of education on REC% is also positive in the study of 12 EU countries conducted by Marra and Colantonio (2021). They conclude that a mix between education and public awareness about environmental issues is key to renewable energy deployment.

## Conclusions

This study analyzed how economic and social factors affected the share of REC in the EU over the period 2001–2015. The results obtained confirmed that, in order to adequately identify which are the main driving factors of REC, regions and countries have to be examined by taking into consideration their particularities, as the same factor may have different impacts depending on the country or region examined.

This study contributed to the extant literature on REC in two main ways. First, it incorporates not only economic but also social factors to the analysis. A large body of the literature on the driving forces of REC includes economic variables. However, there is a lack of evidence on the impact of social factors, especially in high-income countries. We examine the impact of three social factors: education, life expectancy and governance. Second, it compares the results for the EU with those obtained at the world level to identify differential features.

Concerning the two types of factors distinguished, we found that economic factors have a negative effect on REC while social factors have a positive impact in the EU. This means that to facilitate energy transition the EU is called to affect social aspects. In particular, efforts to improve the institutional framework and governance should be conducted. Additionally, the EU should carry out programs to raise awareness of the importance of the use of clean and renewable energy and to promote energy efficiency.

We found that the impact of some factors was different at the EU level and at the world level. Thus, education and life expectancy exert a positive effect on RE consumption in the EU while the impact is negative at the world level. This can be explained, at least in part, by the high level of development of the EU countries compared to the world average. Given the differences in the impact of some the factors examined, national governments should adapt their energy policy to the specific features of their countries. For instance, in the case of developed and developing countries efforts should focus on reducing energy poverty and attracting foreign investments in RE infrastructures.

To conclude, we can affirm that the deployment of RE is influenced by a wide range of factors. In this paper we found that social aspects play a key role in the uptake of RE consumption in the EU. Therefore, it is necessary to adopt a holistic approach that includes not only economic but also social aspects in order to foster the consumption of RE. In this sense, EU governments could implement programs in the different levels of the education systems (schools and universities) aimed at developing knowledge, skills and attitudes to raise environmental awareness and encourage changes in energy use.

## Data Availability

All data generated or analyzed during this study has its sources of public access informed or is included in this published article.

## Appendix

Table

**Table 9:** Country list of empirical econometric models

Afghanistan	Canada	Georgia	Lao PDR	Nigeria	St. Lucia
Albania	Central African Republic	Germany	Latvia	Norway	Vincent and the Grenadines
Algeria	Chad	Ghana	Lebanon	Oman	Sudan
Angola	Chile	Greece	Lesotho	Pakistan	Sweden
Antigua and Barbuda	China	Grenada	Liberia	Palau	Switzerland
Argentina	Colombia	Guatemala	Libya	Panama	Tajikistan
Armenia	Comoros	Guinea	Lithuania	Papua New Guinea	Tanzania
Australia	Congo, Dem. Rep	Guinea-Bissau	Luxembourg	Paraguay	Thailand
Austria	Congo, Rep	Guyana	Madagascar	Peru	Timor-Leste
Azerbaijan	Costa Rica	Haiti	Malawi	Philippines	Togo
Bahamas, The	Cote d'Ivoire	Honduras	Malaysia	Poland	Tonga
Bahrain	Croatia	Hong Kong SAR, China	Maldives	Portugal	Tunisia
Bangladesh	Cyprus	Hungary	Mali	Qatar	Turkey
Barbados	Czech Republic	Iceland	Malta	Romania	Turkmenistan
Belarus	Denmark	India	Mauritania	Russian Federation	Uganda
Belgium	Dominica	Indonesia	Mauritius	Rwanda	Ukraine
Belize	Dominican Republic	Iran, Islamic Rep	Mexico	Samoa	United Arab Emirates
Benin	Ecuador	Iraq	Micronesia, Fed. Sts	Saudi Arabia	United Kingdom
Bhutan	Egypt, Arab Rep	Ireland	Moldova	Senegal	USA
Bolivia	El Salvador	Israel	Mongolia	Serbia	Uruguay
Bosnia and Herzegovina	Equatorial Guinea	Italy	Montenegro	Seychelles	Uzbekistan
Botswana	Eritrea	Jamaica	Morocco	Sierra Leone	Vanuatu
Brazil	Estonia	Japan	Mozambique	Singapore	Venezuela, RB
Brunei Darussalam	Eswatini	Jordan	Myanmar	Slovak Republic	Vietnam
Bulgaria	Ethiopia	Kazakhstan	Namibia	Slovenia	Zambia
Burkina Faso	Fiji	Kenya	Nepal	Solomon Islands	Zimbabwe
Burundi	Finland	Kiribati	Netherlands	South Africa	
Cabo Verde	France	Korea, Rep	New Zealand	Spain	
Cambodia	Gabon	Kuwait	Nicaragua	Sri Lanka	
Cameroon	Gambia, The	Kyrgyz Republic	Niger	St. Kitts and Nevis	

9
